# P-687. Respiratory Syncytial Virus Infection in Patients with Multiple Myeloma

**DOI:** 10.1093/ofid/ofaf695.900

**Published:** 2026-01-11

**Authors:** Risa Fuller, Ian Wright, Samantha E Jacobs, Meenakshi M Rana, Emily Baneman

**Affiliations:** Icahn School of Medicine at Mount Sinai, New York, NY; Icahn School of Medicine at Mount Sinai, New York, NY; Icahn School of Medicine at Mount Sinai, New York, NY; Icahn School of Medicine at Mount Sinai, New York, NY; Icahn School of Medicine at Mount Sinai, New York, NY

## Abstract

**Background:**

The impact of respiratory syncytial virus (RSV) infection in multiple myeloma (MM) patients is not well-defined. We describe the epidemiology and clinical manifestations of RSV in patients with MM as well as risk factors for lower respiratory tract infection (LRTI).

**Table 1:**
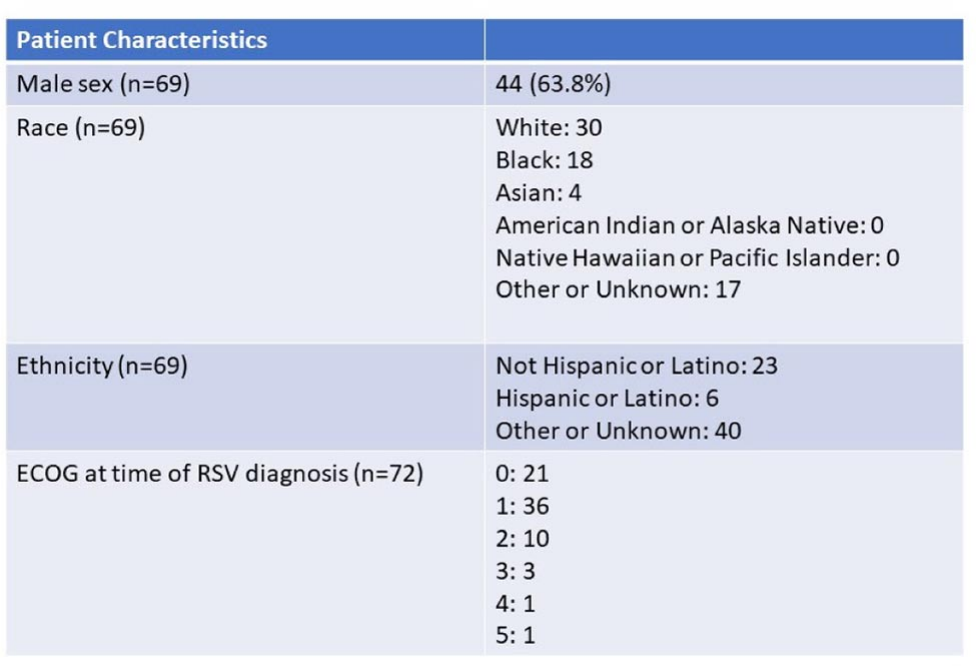
Patient Characteristics

**Table 2:**
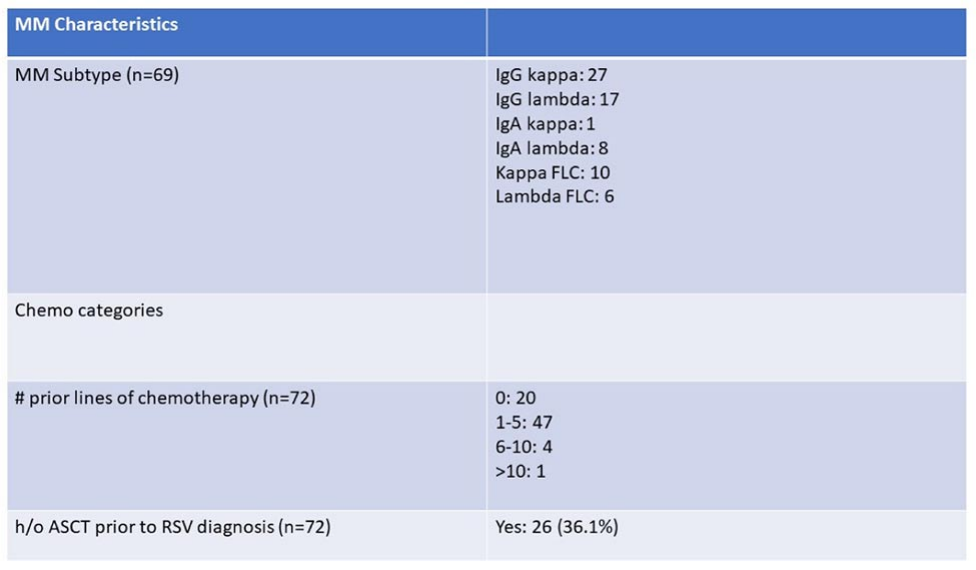
Multiple Myeloma Characteristics

**Methods:**

We queried our electronic health record and identified 72 episodes of RSV infection in 69 unique patients with MM from January 2015 to January 2024.

**Table 3:**
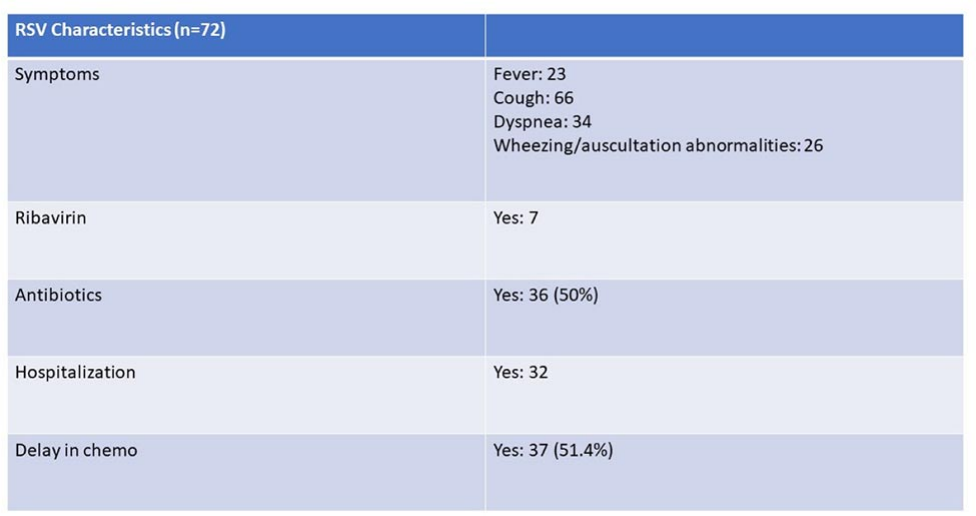
RSV Characteristics

**Table 4:**
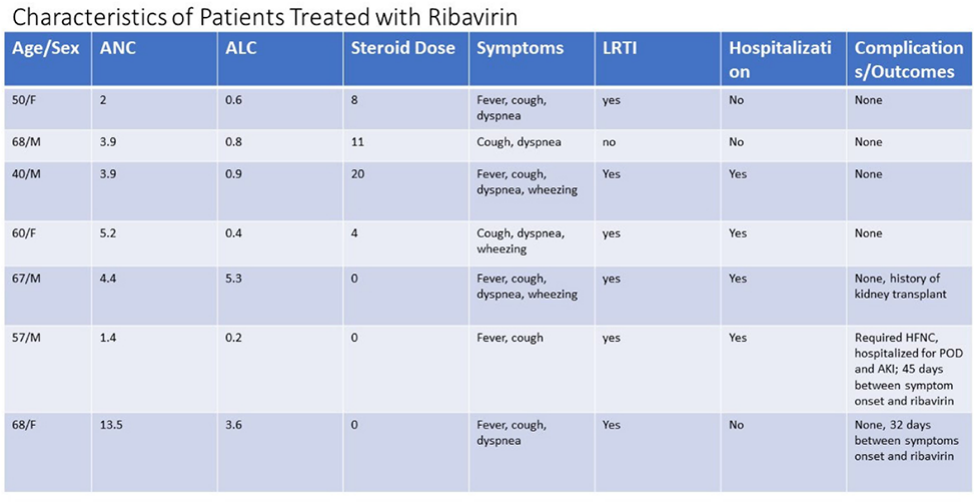
Characteristics of Patients Treated with Ribavirin

**Results:**

The population was predominantly male (64%) and elderly, with median age 68 years (range 40-84). 26 (36%) patients had a history of autologous stem cell transplant and median number of lines of prior MM therapy was 2 (range 0-12). Presenting symptoms were cough (92%), dyspnea (47%), wheezing (36%), and fever (32%). 37 (51%) patients had LRTI and 32 (44%) patients were hospitalized at the time of or soon after RSV diagnosis. Half of the cases were treated with antibiotics and 7 patients received ribavirin. Chemotherapy was delayed in 37 (51.4%) cases. Two patients died within 30 days of infection, neither attributable to RSV. An univariable analysis identified absolute neutrophil count (ANC) and steroid dose within prior 30 days to be associated with LRTI. In the subsequent multivariable model, after adjusting for steroid dose, higher ANC was significantly associated with LRTI (OR = 1.269, 95% CI: [1.019, 1.580], p = 0.033). There was also a trend toward increased LRTI risk with higher steroid dose (OR = 1.036, 95% CI: [0.999, 1.074], p = 0.055), however, these findings should be interpreted with caution due to the limited sample size.

**Conclusion:**

Overall, we found that MM patients commonly had LRTI but did not have attributable mortality from RSV, though infection resulted in a delay in anti-myeloma therapy most of the time. Future trials should assess the impact of RSV vaccines and/or treatments on oncologic outcomes in addition to RSV-related morbidity. Furthermore, half of the patients in our cohort received antibiotics without clear evidence of a bacterial pathogen, representing an opportunity for improved antimicrobial stewardship.

**Disclosures:**

Emily Baneman, MD, Merck: Grant/Research Support

